# Suppressing measurement uncertainty in an inhomogeneous spin star system

**DOI:** 10.1038/s41598-021-02045-0

**Published:** 2021-11-22

**Authors:** Saeed Haddadi, Mehrdad Ghominejad, Ahmad Akhound, Mohammad Reza Pourkarimi

**Affiliations:** 1 Faculty of Physics, Semnan University, P.O.Box 35195-363, Semnan, Iran; 2 Department of Physics, Payame Noor University, P.O.Box 19395-3697, Tehran, Iran; 3 Department of Physics, Salman Farsi University of Kazerun, Kazerun, Iran

**Keywords:** Information theory and computation, Statistical physics, thermodynamics and nonlinear dynamics

## Abstract

The uncertainty principle is known as a foundational element of quantum theory, providing a striking lower bound to quantify our prediction for the measured result of two incompatible observables. In this work, we study the thermal evolution of the entropic uncertainty bound in the presence of quantum memory for an inhomogeneous four-qubit spin-star system that is in the thermal regime. Intriguingly, our results show that the entropic uncertainty bound can be controlled and suppressed by adjusting the inhomogeneity parameter of the system.

## Introduction

The uncertainty principle is undoubtedly one of the most fundamental aspects of quantum mechanics introduced by Heisenberg^1^. The first uncertainty relation for the position ${\hat{x}}$ and the momentum ${\hat{p}}$ was formulated by Kennard as $\Delta {\hat{x}} \Delta {\hat{p}}\ge \hbar /2$^2^. Literally, Heisenberg’s uncertainty principle states that two arbitrary incompatible observables *Q* and *R* cannot be measured accurately at the same time. Later, Robertson^3^ proposed an inequality as $\Delta Q \Delta R\ge |\langle [Q, R]\rangle |/2$ where $\Delta {\mathscr {X}}=\sqrt{\langle {\mathscr {X}}^2\rangle -\langle {\mathscr {X}}\rangle ^2}$ is the standard deviation with ${\mathscr {X}}\in \{Q,R\}$, $\langle {\mathscr {X}}\rangle$ is the mean value of operator ${\mathscr {X}},$ and $[Q, R]=QR-RQ$ is the commutator. In the recent decade, the outstanding achievement accomplished by many authors is a connection between the uncertainty relation and the information theory that is well known as the entropic uncertainty relation (EUR). Deutsch^4^, Kraus^5^, Maassen and Uffink^6^ are researchers who have been pioneers in this subject. Formally, the EUR is defined as $H(Q)+H(R)\ge -\log _{2}c$ where $H(Q)=-\sum _{i}q_{i}\log _{2} q_{i}$ and $H(R)=-\sum _{j}r_{j}\log _{2} r_{j}$ are the Shannon entropy of the probabilities of observables *Q* and *R* measurement results, respectively. The complementarity parameter $c=\max _{i,j}\{|\langle q_{i}|r_{j}\rangle |^{2}\}$ is the maximal overlap of *Q* and *R* with $|q_{i}\rangle$ and $|r_{j}\rangle$ being the eigenstates of *Q* and *R*, respectively.

Nevertheless, a new kind of the EUR in the presence of quantum memory (EUR-QM) has been presented by Berta et al.^7^ in which two players, Alice and Bob, play an uncertainty game. In this game, Bob prepares a correlated two-particle state $\rho ^{AB}$ and he then sends particle *A* to Alice which is correlated to his memory particle *B*. After that, Alice measures her particle with respect to operator *Q* or *R* and announces to Bob the result of her choice. Hence, Bob can guess and minimize his uncertainty based on Alice’s measurement result. This new uncertainty relation can be written as

1 $$\begin{aligned} S(Q|B)+S(R|B) \ge -\log _{2} c+S(A|B), \end{aligned}$$

where $S(A|B)=S(\rho ^{AB})-S(\rho ^{B})$ being the conditional von Neumann entropy of $\rho ^{AB}$ with $\rho ^{B}=tr_{A}(\rho ^{AB})$ and $S(\rho )=-tr(\rho \log _{2}\rho )$ is the von Neumann entropy. Also, $S(Q|B)=S\left( \rho ^{Q B}\right) -S\left( \rho ^{B}\right)$ and $S(R|B)=S\left( \rho ^{R B}\right) -S\left( \rho ^{B}\right)$ are the conditional von Neumann entropies of the post-measurement states $\rho ^{Q B}=\sum _{i}(| q_{i}\rangle _{A}\langle q_{i}| \otimes {\mathbb {I}}_{B}) \rho ^{A B}(| q_{i}\rangle _{A}\langle q_{i}| \otimes {\mathbb {I}}_{B})$ and $\rho ^{R B}=\sum _{j}(| r_{j}\rangle _{A}\langle r_{j}| \otimes {\mathbb {I}}_{B}) \rho ^{A B}(| r_{j}\rangle _{A}\langle r_{j}| \otimes {\mathbb {I}}_{B})$ after the quantum system *A* is measured, and ${\mathbb {I}}_{B}$ being the identity operator.

Till date, the EUR-QM has been the topic of much work^8–48^ and many attempts have been made to tighten this inequality^49–60^. Moreover, many efforts have been made to generalize the various entropic uncertainty relations to more than two measurements^61–67^. Amongst these encouraging efforts, one can refer to the result obtained by Adabi et al.^53^. In 2016, they proposed a tighter bound which has an extra term, comparing to the inequality (1), viz

2 $$\begin{aligned} S(Q|B)+S(R|B) \ge -\log _{2} c+S(A|B)+\max \{0, \kappa \}, \end{aligned}$$

with $\kappa =I(A:B)-[I(Q:B)+I(R : B)]$ where $I(A:B)=S(\rho ^{A})+S(\rho ^{B})-S(\rho ^{AB})$ is mutual information, $I({\mathscr {X}}:B)=S\left( \rho ^{B}\right) -\sum _{x} p_{x} S\left( \rho _{x}^{B}\right)$ is Holevo quantity, $\rho _x^{B}=tr_{A}(\Pi _{x}^{A}\rho ^{AB}\Pi _x^{A})/p_x$ is the post-measurement state of Bob after measuring of ${\mathscr {X}}$ by Alice, and $p_x=tr_{AB}(\Pi _{x}^{A}\rho ^{AB}\Pi _x^{A})$ is the probability of *x*th outcome. Note that the tightness of the uncertainty relation means that the difference between the uncertainty and its bound is the smallest value. In this work, we consider the right-hand side of the inequality (2) as an entropic uncertainty bound, i.e.,

3 $$\begin{aligned} U_{b}(\rho ^{AB})\equiv -\log _{2} c+S(A|B)+\max \{0, \kappa \}. \end{aligned}$$

In recent decades, many researchers have been interested in studying spin systems because of their application in quantum information^68–71^. The spin chain is one of the most popular spin systems in which spins interact with their neighbors, well known as the Heisenberg model, which has been sufficiently reviewed by many authors so far^72–84^. Motivated by this, we study another kind of spin system known as a spin-star system for which spins cannot interact with each other directly, and a central spin is responsible for interacting with the other spins. Historically, the study of the spin-star system started in 2004 when Hutton and Bose^85^ investigated the interesting properties of a physical system that a central spin interacts with the outer ones. In literature, lots of researches have been devoted to investigating quantum correlations in the spin-star system^86–96^, e.g., Anzà et al.^93^ investigated tripartite thermal correlations in an inhomogeneous spin-star system using concurrence and tripartite negativity criteria. The authors examined the dependence of such quantum correlations on the homogeneity and inhomogeneity of the interactions, and they found some interesting differences between the tripartite negativity and concurrence. Moreover, Militello and Messina^94^ examined the tripartite thermal entanglement in an inhomogeneous spin-star network with three external spins. From a practical point of view, the spin-star system can be realized in many solid state systems, such as the nitrogen-vacancy centre in diamond^97,98^ and the semiconductor quantum dot^99–101^ (see recent paper^102^ and references therein for a detailed study on the spin-star system). However, to the best of our knowledge, nobody has previously examined the entropic uncertainty for a spin-star system up to now. Because of the lack of such exploring, we are then motivated to investigate the entropic uncertainty bound in a four-qubit spin-star system and finding a suitable parameter to control the entropic uncertainty of the system. Hence, this paper is prepared as follows. In "Physical scenario", we consider a four-qubit spin-star system where three outer qubits are coupled to the central one with different strengths, and in "Results and discussion", thermal entropic uncertainty bound in different situations is analyzed. Finally, we discuss our results and present some conclusive remarks subsequently.

## Physical scenario

**Figure 1:**
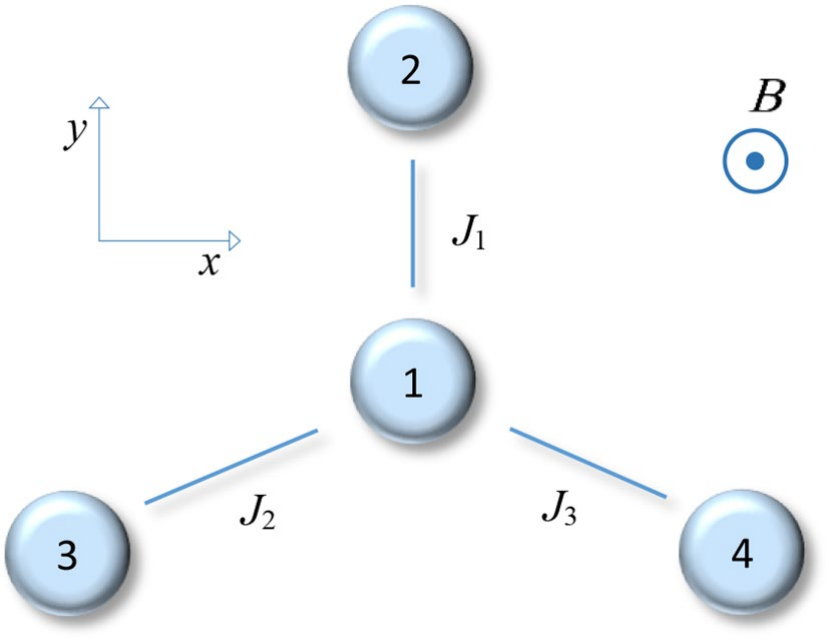
A schematic diagram of a four-qubit spin-star system. The qubits labelled 2–4 are coupled to the central one with $J_{1}$, $J_{2}$, and $J_{3}$ which are the coupling constants, respectively. The external magnetic field ***B*** is perpendicular to the plane where the four qubits lie.

This section considers a four-qubit spin-star system for which the schematic diagram of such a quantum system is sketched in Fig. 1. The total Hamiltonian of the mentioned system is given by (setting $\hbar =1$)

4 $$\begin{aligned} \mathbf{H} =\sum _{i=1}^{3} J_{i}\left( \sigma ^{+}_{1} \sigma ^{-}_{i+1}+\sigma ^{-}_{1} \sigma ^{+}_{i+1}\right) + {\varvec{{B}}} \sum _{i=1}^{4} \sigma ^{z}_{i}, \end{aligned}$$

where $J_{1}$, $J_{2}$, and $J_{3}$ are the coupling constants of the central qubit with the outer qubits 2, 3, and 4, respectively. Besides, ***B*** indicates the magnitude of the external magnetic field, $\sigma _{i}^{z}(i=1,2,3,4)$ is the Pauli operator in the *z*-direction for *i*th qubit, and $\sigma ^{+}_{i}$ as well as $\sigma ^{-}_{i}$ are the ladder operators.

Let us assume that a typical system reaches thermodynamical equilibrium. So, its density operator can be described by the thermal state as

5 $$\begin{aligned} \rho _{T}=\frac{1}{{\mathscr {Z}}}\exp (-\mathbf{H} /\kappa _{B}T)=\frac{1}{{\mathscr {Z}}}\sum _{i}\exp (-E_{i}/\kappa _{B}T)|\psi _{i}\rangle \langle \psi _{i}|, \end{aligned}$$

where ${\mathscr {Z}}=tr[\exp ( - \mathbf{H} /\kappa _{B}T)]$ is the system partition function with $\kappa _{B}$ as the Boltzmann constant, considered $\kappa _{B}=1$ by us, as it is used in the natural unit system. Moreover, $E_i$ and $|\psi _{i}\rangle$ are the eigenvalues and the eigenstates of the total Hamiltonian, respectively.

Now, we consider an inhomogeneous Hamiltonian for which $J_1=J_3=J$ and $J_2=xJ$, where *x* is a suitable dimensionless inhomogeneity parameter. It is clear that the homogeneous Hamiltonian is obtained for $x=1$. Having known the Hamiltonian (4) and assuming inhomogeneity, the results of the diagonalization of $\mathbf{H}$ are reported in “Methods”. Then, by using Eq. (5) and through taking partial traces over the outer two qubits for a four-qubit thermal state, the reduced density matrices in the standard basis $\{|00\rangle$, $|01\rangle$, $|10\rangle , |11\rangle \}$ with $A=1$ and $B=2,3,4$ are given in “Methods”. It is quite obvious that due to the inhomogeneity, $\rho ^{12}_T=\rho ^{14}_T \ne \rho ^{13}_T$, so let’s call $\rho _{T,1}=\rho ^{12}_T=\rho ^{14}_T$ as case 1 and $\rho _{T,2}=\rho ^{13}_T$ as case 2, henceforth.

## Results and discussion

Here, we would like to investigate the thermal evolution of the entropic uncertainty bound by considering two thermal quantum states ($\rho _{T,1}$ and $\rho _{T,2}$). In each of the related subsections, we will explicitly present and discuss the analytical results.

### Model with inhomogeneity of case 1

Based upon the results of the previous section, we can now analyze the thermal evolution of the entropic uncertainty bound for the quantum state $\rho _{T,1}$ in this section. In this scenario, Bob prepares a correlated two-qubit state $\rho _{T,1}$ and then he sends one qubit to Alice and keeps the other one as a quantum memory in his hand. Herein, without loss of the generality, we consider the situation where Alice measures one of the two observables $Q=\sigma ^x$ or $R=\sigma ^z$ where $\sigma ^x$ and $\sigma ^z$ are the Pauli operators and then, we will obtain the complementarity parameter as $c=1/2$. The thermal reduced density matrix $\rho ^{12}_T=\rho ^{14}_T=tr_{34}(\rho _T)=tr_{23}(\rho _T)$ is obtained as

6 $$\begin{aligned} \rho _{T,1}=\rho ^{12}_T=\rho ^{14}_T=\left( \begin{array}{cccc} v &{} 0 &{} 0 &{} 0 \\ 0 &{} u^{+} &{} w &{} 0 \\ 0 &{} w &{} u^{-} &{} 0 \\ 0 &{} 0 &{} 0 &{} q \end{array}\right) . \end{aligned}$$

Note that, the density matrix components are reported in “Methods”. Obviously now, by substituting the elements of the thermal reduced density matrix (6) into Eq. (3), one can obtain the analytical expression of the entropic uncertainty bound as what follows

7 $$\begin{aligned} U_{b}(\rho _{T,1})=\Delta _{T,1}+\max \{0, \Theta _{T,1}\}, \end{aligned}$$

where

8 $$\begin{aligned} \Delta _{T,1}=1-H_{2}(v+u^{-})-\sum _{i} \tau _{i} \log _2 \tau _i, \end{aligned}$$

and

9 $$\begin{aligned} \Theta _{T,1}=-H_{2}(v+u^{-})+H_{2} \left(\frac{1+k}{2} \right) -\sum _{i} \rho _{T,1 (ii)} \log _2 \rho _{T,1 (ii)} +\sum _{i} \tau _{i} \log _2 \tau _i, \end{aligned}$$

where $\tau _{i}$’s are the eigenvalues of $\rho _{T,1}$ and $k=\sqrt{4|w|^{2}+[1-2(q+u^{+})]^{2}}$. Above, $S(\rho ^{B}_{T,1})=H_{2}(v+u^{-})$ is the von Neumann entropy of quantum memory and $H_{2}(\varepsilon )= -\epsilon \log _{2}\varepsilon -(1-\varepsilon )\log _{2}(1-\varepsilon )$ for any $\varepsilon \in [0,1]$ is the binary Shannon entropy function.

Up to now, we have analytically derived the entropic uncertainty bound for a quantum state $\rho _{T,1}$. However, our results show that to possess an exact solution for that, depends on the different parameters of the system under study. Hence, let us analyze the entropic uncertainty bound as functions of the temperature and the system parameters.

**Figure 2:**
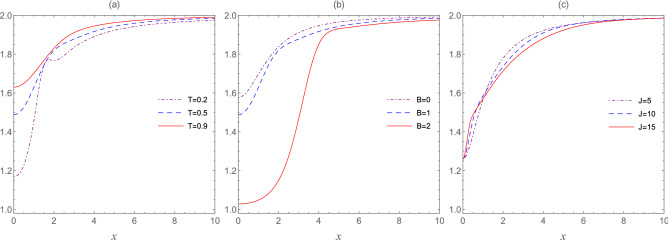
The entropic uncertainty bound $U_{b}(\rho _{T,1})$ as a function of the inhomogeneity parameter *x* for fixed values of the temperature *T*, external magnetic field ***B***, and the coupling constant *J*. Graph (**a**) ***B*** = 1; $J=1$, graph (**b**) $T=0.5$; $J=1$, and graph (**c**) $T=0.5$; ***B*** = 1.

Figure 2 shows the thermal evolution of the entropic uncertainty bound (7) as a function of the inhomogeneity parameter (*x*) for specific values of the temperature (*T*), external magnetic field (***B***), and the coupling constant (*J*) between the central spin and the peripheral ones. Following, Fig. 2a shows that the entropic uncertainty bound of $\rho _{T,1}$ grows with increasing the temperature, and hence, Bob’s uncertainty about Alice’s measurement outcome increases. Of course, this is not surprising, as the increasing temperature can reduce quantum correlations, leading to an increase in measurement uncertainty of incompatible observables. It is quite clear that the physical reason for increasing the uncertainty with respect to temperature, is the high degree of mixedness. Compared to temperature, here the role of the external magnetic field is quite constructive to predict the results of Alice’s measurement by Bob, as seen from Fig. 2b. The role of the coupling constant, as shown in Fig. 2c, is quite complicated. This means that the entropic uncertainty bound for $x<1$ increases with increasing the coupling constant, but for $x>1$ the event is different. In fact, when *x* is much greater than 1, $J_{2} = xJ$ is much greater than $J_{1} = J_{3} = J$, and so, roughly speaking, qubits 2 and 4 can be considered almost decoupled, and then separable. For this reason, the increase in the inhomogeneity parameter notably leads to reducing Bob’s ability to predict Alice’s measurement outcome, as can be seen from all plots. It is worth noting that for $x=1$, the system under study is a homogeneous model.

**Figure 3:**
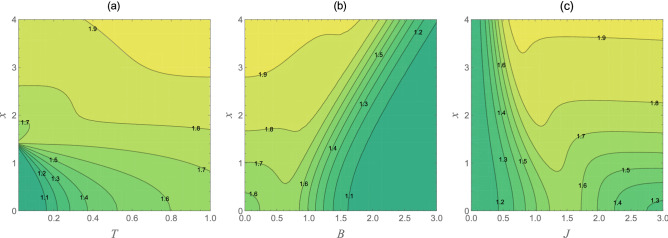
The entropic uncertainty bound $U_{b}(\rho _{T,1})$ as a function of the inhomogeneity parameter *x* and different values of the temperature *T*, external magnetic field ***B***, and the coupling constant *J*. Graph (**a**) ***B*** = 1; $J=1$, graph (**b**) $T=0.5$; $J=1$, and graph (**c**) $T=0.5$; ***B*** = 1.

For more clarification, we have drawn contour diagrams of the entropic uncertainty bound as functions of the inhomogeneity parameter *x* and different values of the temperature, external magnetic field, and the coupling constant in Fig. 3. As depicted in Fig. 3a, the entropic uncertainty bound increases dramatically with the growth in the inhomogeneity parameter *x* and temperature value, with ***B*** = 1 and $J=1$, which is obviously in accordance with what mentioned above. In Fig. 3b, the entropic uncertainty bound as functions of *x* and ***B*** has been plotted. It directly shows that the smaller *x* and the greater ***B*** can both induce Bob’s uncertainty about Alice’s measurement result to become less, which is desired in practical quantum information processing. Let us now turn to focus on the influence of the inhomogeneity parameter *x* and the strength of the coupling parameter on the entropic uncertainty bound, as shown in Fig. 3c. At a fixed temperature and ***B*** = 1, in the $0<J<1/2$ area, the entropic uncertainty bound is somewhat insensitive to the inhomogeneity parameter variations. However, in the $1/2\le J\le 3$ area, the inhomogeneity parameter appears to be a dominant factor.

### Model with inhomogeneity of case 2

According to the previous analysis, we can obtain the analytical expression of the entropic uncertainty bound for the quantum state $\rho _{T,2}$. The thermal reduced density matrix $\rho ^{13}_T=tr_{24}(\rho _T)$ is taken as follows

10 $$\begin{aligned} \rho _{T,2}=\rho ^{13}_T=\left( \begin{array}{cccc} \vartheta &{} 0 &{} 0 &{} 0 \\ 0 &{} \mu ^{+} &{} \nu &{} 0 \\ 0 &{} \nu &{} \mu ^{-} &{} 0 \\ 0 &{} 0 &{} 0 &{} \xi \end{array}\right) , \end{aligned}$$

where the non-zero elements are given again in “Methods”. By substituting the elements of the thermal reduced density matrix (10) into Eq. (3), it is again easy to see that

11 $$\begin{aligned} U_{b}(\rho _{T,2})=\Delta _{T,2}+\max \{0, \Theta _{T,2}\}, \end{aligned}$$

where

12 $$\begin{aligned} \Delta _{T,2}=1-H_{2}(\vartheta +\mu ^{-})-\sum _{i} \chi _{i} \log _2 \chi _i, \end{aligned}$$

and

13 $$\begin{aligned} \Theta _{T,2}=-H_{2}(\vartheta +\mu ^{-})+H_{2}(\frac{1+\varsigma }{2})-\sum _{i} \rho _{T,2 (ii)} \log _2 \rho _{T,2 (ii)} +\sum _{i} \chi _{i} \log _2 \chi _i, \end{aligned}$$

where $\chi _{i}$’s are the eigenvalues of $\rho _{T,2}$ and $\varsigma =\sqrt{4|\nu |^{2}+[1-2(\xi +\mu ^{+})]^{2}}$. Here, $S(\rho ^{B}_{T,2})=H_{2}(\vartheta +\mu ^{-})$ denotes the von Neumann entropy of quantum memory.

**Figure 4:**
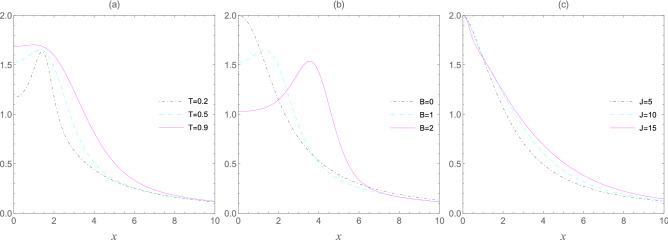
The entropic uncertainty bound $U_{b}(\rho _{T,2})$ as a function of the inhomogeneity parameter *x* for fixed values of the temperature *T*, external magnetic field ***B***, and the coupling constant *J*. Graph (**a**) ***B*** = 1; $J=1$, graph (**b**) $T=0.5$; $J=1$, and graph (**c**) $T=0.5$; ***B*** = 1.

In Fig. 4, we first examine the effect of temperature on the entropic uncertainty of interest and draw the thermal evolution of the entropic uncertainty bound $U_{b}(\rho _{T,2})$ as functions of inhomogeneity parameter *x* and fixed values of *T* in Fig. 4a. As the previous case, one can observe that the entropic uncertainty bound increases by growing the temperature. On the other hand, in analyzing the effects of the inhomogeneity parameter *x* on the entropic uncertainty, we observe that the entropic uncertainty grows when the value of *x* enhances at lower temperatures, and after passing a peak, it becomes smaller and quickly achieves a steady value for the larger amounts of *x*. As plotted in Fig. 4b, for ***B***=1 and ***B***=2, it is obvious that the entropic uncertainty bound is firstly increased and then reduces as *x* raises. However, in the absence of an external magnetic field (***B***=0), the uncertainty bound monotonously decreases when *x* grows. In order to further probe the relationship between the uncertainty bound and the inhomogeneity *x* as well as the coupling coefficient *J*, we have drawn the uncertainty bound as a function of *x* for three values of *J*, as displayed in Fig. 4c. At fixed temperature and ***B***=1, in the $0< x < 1$ area, while *x* is much smaller than 1, $J_2 = xJ$ is much smaller than $J_1 = J_3 = J$, and hence the qubit 3 can be considered almost decoupled, and then the uncertainty bound is maximal for $x=0$. Nevertheless, for *x* greater than 1, the coupling between the central qubit and the qubit 3 becomes more robust than the other two, and hence the measurement uncertainty is significantly suppressed.

**Figure 5:**
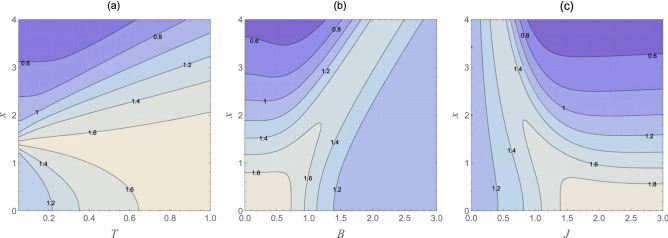
The entropic uncertainty bound $U_{b}(\rho _{T,2})$ as a function of the inhomogeneity parameter *x* and different values of the temperature *T*, external magnetic field ***B***, and the coupling constant *J*. Graph (**a**) ***B*** = 1; $J=1$, graph (**b**) $T=0.5$; $J=1$, and graph (**c**) $T=0.5$; ***B*** = 1.

To further study the relationship between the uncertainty bound and the inhomogeneity *x* as well as the other parameters of the thermal system, we have drawn the contour diagrams of the uncertainty bound as functions of *T*, ***B***, and *J* in plots (a) to (c) respectively, as shown in Fig. 5. One can readily observe that the uncertainty bound decreases with the reducing *T*, but the role of the external magnetic field ***B*** and the coupling coefficient *J* seem to be somewhat complex. Hence, we can see that these achieved results are well in agreement with the report we made earlier in Fig. 4. In short, regardless of the temperature values and the values of ***B*** and *J*, the greater inhomogeneity parameter *x* can induce a smaller uncertainty bound, and vice versa. This result apparently implies that the maximum symmetry in the four-qubit spin-star system does not correspond to the minimum uncertainties between all its parts, i.e., the uncertainties between qubits 1–2, 1–3, and 1–4.

### Comparison of case 1 with case 2

After reviewing the analytical and numerical results, let us now compare the thermal evolution of the entropic uncertainty bound in two cases. From the outlook of quantitative description of the entropic uncertainty bound, we need to state that in the thermal state $\rho _{T,1}$, regardless of the temperature, ***B***, and *J*, the uncertainty bound increases and reach a maximum fixed value (saturated value) when the inhomogeneity *x* grows ($U_{b}(\rho _{T,1})\simeq 2$). Therefore, in this case, Bob’s information about the result of Alice’s measurement is reduced rapidly. However, in the case of the thermal state $\rho _{T,2}$, the uncertainty bound comes near to zero when the inhomogeneity *x* increases. The physical reason is that as long as the inhomogeneity *x* grows, the coupling between the central qubit and the qubit 3 becomes stronger than the other two, and therefore $U_{b}(\rho _{T,2})\rightarrow 0$ while $U_{b}(\rho _{T,1})\rightarrow 2$. It is worth noting that in this situation ($\rho _{T,2}$), Bob’s uncertainty about Alice’s measurement outcome remarkably decreases. Hence, we find that the uncertainty bound can be controlled by manipulating the inhomogeneity parameter and then Bob can accurately guess Alice’s measurement outcome.

### Physical interpretation and possible applications

In this work, we have examined the features of thermal entropic uncertainties in the presence of inhomogeneity since in real conditions the construction of a system with ideally homogeneous interactions could be much challenging. Accordingly, we have shown that the measurement uncertainty of incompatible observables is controllable by regulating the inhomogeneity parameter. This implies the idea that the thermal uncertainties mediated by the central spin can be remarkably controlled by a certain absence of homogeneity that could describe a more realistic situation, even if the degree of inhomogeneity is high. Hence, our result may be advantageous for practical quantum information processing, and it is physically realizable in different arrays of interacting particles designed for quantum computing^103^. Notably, controlling the entropic uncertainty has some versatile applications in practical quantum tasks including, quantum cryptography^104^, entanglement witness^105^, quantum key distribution^106^, quantum metrology^107^, quantum teleportation^108^, quantum steering^109^, and so on^110^. Specifically, measurement of uncertainty in a spin-star system can be experimentally realized by molecular nanomagnets, NMR molecules, superconducting spins, and coupled microcavities^111^.

## Conclusions and remarks

To conclude, we have studied the entropic uncertainty bound in a spin-star system with three peripheral qubits, all affected by an external magnetic field. The interaction with the central qubit is responsible for establishing tripartite correlations between the outer ones, and such correlations remain even if the system is at thermal equilibrium. We have considered an inhomogeneous model, where one of the peripheral qubits is coupled to the central one with a different strength. In the following, we analytically derived the entropic uncertainty bound for the quantum states $\rho _{T,1}$ and $\rho _{T,2}$ and then we investigated the thermal evolution of the entropic uncertainty bound in measuring two incompatible observables under the effect of the temperature *T*, the magnetic field ***B***, the coupling coefficient strength *J*, and the inhomogeneity parameter *x*. A remarkable point is, we found that the entropic uncertainty bound can be improved by changing the inhomogeneity as an efficient control parameter. Therefore, we think that a higher degree of symmetry in the system does not guarantee fewer uncertainties between all its parts. This issue could be highly instructive and available to quantum precision measurement by adjusting proper measurement parameters and conditions.

## Methods

### Eigenvalues and the eigenstates of the Hamiltonian

In this section, we give the eigenvalues $E_{i}$ and the eigenstates $|\psi _{i}\rangle$ of the inhomogeneous Hamiltonian as functions of $x>0, J>0,$ and ***B***. The homogeneous model is achieved for $x=1$. The eigenvalues of the Hamiltonian are

14 $$\begin{aligned}{}&E_{1}=2{\varvec{{B}}}, \quad E_{2}=-2{\varvec{{B}}}, \quad E_{3}^{\pm }=\pm Jx, \quad E_{4}^{\pm }=\pm \frac{J}{2} \left( x+ \sqrt{x^2+8}\right) , \quad E_{5}^{\pm }=\pm \frac{J}{2} \left( x- \sqrt{x^2+8}\right) , \\&E_{6}^{\pm }=2 {\varvec{{B}}}\pm J \sqrt{x^2+2}, \quad E_{7}^{\pm }= -2 {\varvec{{B}}} \pm J \sqrt{x^2+2}, \quad E_{8}=-4{\varvec{{B}}}, \quad E_{9}=4{\varvec{{B}}}, \end{aligned}$$

where the eigenvalues $E_{1}$ and $E_{2}$ are twofold degenerate eigenvalues. The relevant eigenstates are

15 $$\begin{aligned} |\psi _{1}^{a}\rangle =&\frac{1}{M_{1}}\left[ -\frac{1}{x}|0001\rangle -|0010\rangle +\left( \frac{1}{x}+x\right) |0100\rangle \right] , \end{aligned}$$

16 $$\begin{aligned} |\psi _{1}^{b}\rangle =&\frac{1}{\sqrt{x^2+1}}\left( -x|0001\rangle +|0010\rangle \right) , \end{aligned}$$

17 $$\begin{aligned} |\psi _{2}^{a}\rangle =&\frac{1}{M_{1}}\left[ -\frac{1}{x}|1011\rangle -|1101\rangle +\left( \frac{1}{x}+x\right) |1110\rangle \right] , \end{aligned}$$

18 $$\begin{aligned} |\psi _{2}^{b}\rangle =&\frac{1}{\sqrt{x^2+1}}\left( -x|1011\rangle +|1101\rangle \right) , \end{aligned}$$

19 $$\begin{aligned} |\psi _{3}^{\pm }\rangle =&\frac{1}{2}\left[ (|1100\rangle \pm |0110\rangle )-(|1001\rangle \pm |0011\rangle ) \right] , \end{aligned}$$

20 $$\begin{aligned} |\psi _{4}^{\pm }\rangle =&\frac{1}{M_{2}}\big [(|1001\rangle \pm |0011\rangle ) +(|1100\rangle \pm |0110\rangle ) +\frac{\sqrt{x^2+8}-x}{2}(\left| 1010\rangle \pm |0101\rangle \right) \big ], \end{aligned}$$

21 $$\begin{aligned} |\psi _{5}^{\pm }\rangle =&\frac{1}{M_{2}}\big [(|1001\rangle \pm |0011\rangle ) +(|1100\rangle \pm |0110\rangle )-\frac{\sqrt{x^2+8}+x}{2}(\left| 1010\rangle \pm |0101\rangle \right) \big ], \end{aligned}$$

22 $$\begin{aligned} |\psi _{6}^{\pm }\rangle =&\frac{1}{M_{3}}\left[ \sqrt{x^2+2}|1000\rangle \pm (|0001\rangle + x|0010\rangle +|0100\rangle )\right] , \end{aligned}$$

23 $$\begin{aligned} |\psi _{7}^{\pm }\rangle =&\frac{1}{M_{3}}\left[ (|1011\rangle +x|1101\rangle +|1110\rangle )\pm \sqrt{x^2+2}|0111\rangle \right] , \end{aligned}$$

24 $$\begin{aligned} |\psi _{8}\rangle =&|0000\rangle , \end{aligned}$$

25 $$\begin{aligned} |\psi _{9}\rangle =&|1111\rangle , \end{aligned}$$

with

26 $$\begin{aligned} M_{1}^{2}=&\frac{2}{x^{2}}+3+x^{2},\quad M_{2}^{2}=8+ x \left( x-\sqrt{x^2+8}\right) , \quad M_{3}^{2}=2\left( 2+x^{2}\right) . \end{aligned}$$

### Matrix elements of thermal reduced density matrices

Here, we give the matrix elements of the thermal reduced density matrices $\left( \rho ^{12}_T=\rho ^{14}_T \ne \rho ^{13}_T\right)$ for the inhomogeneous Hamiltonian as functions of $x,J>0$, and ***B***. The matrix elements of thermal reduced density matrix $\rho _{T,1}$ read

27 $$\begin{aligned}{}&v= \frac{1}{2{\mathscr {Z}}}\bigg (\cosh [4 \gamma ]+\cosh [x \delta ] +2 \eta ^{-1} e^{-2 \gamma }(\eta +1+(\eta -1) \cosh [\delta \sqrt{\eta } ]) \\&\quad +\cosh \left[ x \delta /2\right] \cosh \left[ \delta \sqrt{\theta } /2\right] -2 \sinh [4 \gamma ] +x \theta ^{-\frac{1}{2}} \sinh \left[ x \delta /2\right] \sinh \left[ \delta \sqrt{\theta } /2\right] \bigg ), \end{aligned}$$

28 $$\begin{aligned}u^{\pm } & = \frac{1}{4 {\mathscr {Z}} \eta \theta }\bigg (4 \cosh [2 \gamma ]\left( 8+9 x^{2}+x^{4}+(\eta +1) \theta \cosh [ \delta \sqrt{\eta }]\right) +2 \eta \theta \left( \cosh [x \delta ]+3 \cosh \left[ x \delta /2\right] \cosh \left[ \delta \sqrt{\theta } /2\right] \right) \\&\quad +x^{3} \sqrt{\theta } \left( \cosh \left[ \delta \alpha _{-}/2\right] - \cosh \left[ \delta \alpha _{+}/2\right] \right) +2 x \sqrt{\theta } \left( \cosh \left[ \delta \alpha _{-}/2\right] - \cosh \left[ \delta \alpha _{+}/2\right] \right) \\&\quad \pm 8\left( 8+9 x^{2}+x^{4}\right) \sinh [2 \gamma ] \sinh ^{2}\left[ \eta \delta /2\right] \bigg ), \end{aligned}$$

29 $$\begin{aligned}q &= \frac{1}{4 {\mathscr {Z}}\eta \theta }\bigg (4 \eta \theta (\sinh [4 \gamma ]+\cosh [4 \gamma ]) +2 \eta \theta \left( \cosh [x \delta ]+\cosh \left[ x \delta /2\right] \cosh \left[ \delta \sqrt{\theta }/2\right] \right) \\&\quad +x^{3} \sqrt{\theta } \left( \cosh \left[ \delta \alpha _{+}/2\right] - \cosh \left[ \delta \alpha _{-}/2\right] \right) +2 x \sqrt{\theta } \left( \cosh \left[ \delta \alpha _{+}/2\right] - \cosh \left[ \delta \alpha _{-}/2\right] \right) \\&\quad +4 \theta (\sinh [2 \gamma ]+\cosh [2 \gamma ])(\eta +1+(\eta -1) \cosh [\delta \sqrt{\eta }]) \bigg ), \end{aligned}$$

30 $$\begin{aligned}&w=\frac{1}{{\mathscr {Z}}}\left( -2 \eta ^{-\frac{1}{2}} \cosh [2 \gamma ] \sinh [\delta \sqrt{\eta }]-4 \theta ^{-\frac{1}{2}} \cosh \left[ x \delta /2\right] \sinh \left[ \delta \sqrt{\theta } /2\right] \right) , \end{aligned}$$

besides, the matrix elements of thermal reduced density matrix $\rho _{T,2}$ read

31 $$\begin{aligned} \vartheta =&\frac{1}{{\mathscr {Z}}}\bigg (e^{-4 \gamma }+\eta ^{-1}\left( e^{-2 \gamma +\delta \sqrt{\eta }}+e^{-2 \gamma -\delta \sqrt{\eta }}+2(\eta -1) e^{-2 \gamma }\right) \\&+\frac{1}{2}\left[ \cosh \left[ \delta \alpha _{+}/2\right] \left( 1-x \theta ^{-\frac{1}{2}}\right) +\cosh \left[ \delta \alpha _{-}/2\right] \left( 1+x \theta ^{-\frac{1}{2}}\right) \right] \bigg ), \end{aligned}$$

32 $$\begin{aligned} \mu ^{\pm }=&\frac{1}{{\mathscr {Z}}} \bigg (\cosh [x \delta ]+2 \eta ^{-1} \cosh [2 \gamma ](1+(\eta -1) \cosh [\delta \sqrt{\eta }]) \\&+\cosh \left[ x \delta /2\right] \cosh \left[ \delta \sqrt{\theta }/2\right] +x \theta ^{-\frac{1}{2}} \sinh \left[ x \delta /2\right] \sinh \left[ \delta \sqrt{\theta }/2\right] \\&\pm 4 \eta ^{-1} \sinh [2 \gamma ] \sinh ^{2}\left[ \delta \sqrt{\eta }/2\right] \bigg ), \end{aligned}$$

33 $$\begin{aligned} \xi =&\frac{1}{2 {\mathscr {Z}} \eta \theta }\bigg (2 \eta \theta (\sinh [4 \gamma ]+ \cosh [4 \gamma ]) +2 \eta \theta \cosh \left[ x \delta /2\right] \cosh \left[ \delta \sqrt{\theta }/2\right] \\&+x^{3} \sqrt{\theta } \left( \cosh \left[ \delta \alpha _{-}/2\right] - \cosh \left[ \delta \alpha _{+}/2\right] \right) +2 x \sqrt{\theta } \left( \cosh \left[ \delta \alpha _{-}/2\right] - \cosh \left[ \delta \alpha _{+}/2\right] \right) \\&+4 \theta (\sinh [2 \gamma ]+\cosh [2 \gamma ])(\eta -1+\cosh [\delta \sqrt{\eta }])\bigg ), \end{aligned}$$

34 $$\begin{aligned} \nu =&\frac{1}{{\mathscr {Z}}} \bigg (-\cosh \left[ \delta \sqrt{\theta }/2\right] \sinh \left[ x \delta /2\right] -\sinh [x \delta ] -2 x \eta ^{-\frac{1}{2}} \cosh [2 \gamma ] \sinh \left[ \delta \sqrt{\eta }\right] \\&-x \theta ^{-\frac{1}{2}} \cosh \left[ x \delta /2\right] \sinh \left[ \delta \sqrt{\theta }/2\right] \bigg ), \end{aligned}$$

with

35 $$\begin{aligned} {\mathscr {Z}}=&2\bigg (\cosh [4 \gamma ]+\cosh [x \delta ]+2 \cosh [2 \gamma ](1+\cosh \left[ \delta \sqrt{\eta }\right] ) +2 \cosh \left[ x \delta /2\right] \cosh \left[ \delta \sqrt{\theta }/2\right] \bigg ), \end{aligned}$$

and

36 $$\begin{aligned} \eta =2+x^{2}, \quad \theta =8+x^{2}, \quad \alpha _{\pm }=x \pm \sqrt{\theta }, \quad \gamma ={\varvec{{B}}}/T, \quad \delta =J/T. \end{aligned}$$

## Data Availability

All data generated or analysed during this study are included in this paper.
